# Reactive oxygen metabolites can be used to differentiate malignant and non-malignant pleural efffusions

**DOI:** 10.4103/1817-1737.65042

**Published:** 2010

**Authors:** Ufuk Cobanoglu, Fuat Sayir, Duygu Mergan

**Affiliations:** *Faculty of Medicine, Department of Thoracic Surgery, Yuzuncu Yil University, Van, Turkey*

**Keywords:** Malignant pleural effusion, non-malignant pleural effusion, reactive oxygen metabolites

## Abstract

**OBJECTIVE:**

Increase in reactive oxygen metabolites (ROM) and free radicals is an important cause of cell injury. In this study, we investigated whether determination of ROM in pleural fluids of patients with malignant and non-malignant pleural effusions can be used as a tumor marker indicating malignant effusions in the differential diagnosis.

**METHODS:**

Sixty subjects with exudative pleural effusion and 25 healthy individuals as the control group were included in the study. Of the subjects with pleural effusion, 50% were malignant and 50% were non-malignant. ROM was studied in the pleural fluids and sera of the subjects with pleural effusion and in the sera of those in the control group. The ROM values of smokers and non-smokers were compared in each group. The Student’s *t*-test and the Mann-Whitney U test were used in order to detect differences between groups for descriptive statistics in terms of pointed features. The statistical significance level was set at 5% in computations, and the computations were made using the SPSS (ver.13) statistical package program

**RESULTS:**

It was determined that the difference between the ROM values of subjects with malignant and non-malign pleural effusions and the sera of the control group was significant in the malignant group compared to both groups (*P* = 0.0001), and the sera ROM values of patients with non-malignant pleural effusion were significant compared to the control group (*P* = 0.0001), and the ROM values of smokers were significant compared to non-smokers in each of the three groups (*P* = 0.0001).

**CONCLUSION:**

These findings indicate that sera ROM levels are increased considerably in patients with exudative effusions compared to that of the control group. This condition can be instructive in terms of serum ROM value being suggestive of exudative effusion in patients with effusions. Furthermore, the detection of pleural ROM values being significantly higher in subjects with malignant pleural effusions compared to non-malignant subjects suggests that ROM can be used as a tumor marker in the differential diagnosis of pleural effusions of unknown origin.

Pleural effusions are mirrors of systemic diseases. Diseases of every system or organ can affect the pleura. For this reason, in the evaluation of pleural effusions, not only pulmonary or pleural diseases, but also pathologies below the diaphragm, systemic diseases and lymphatic system diseases must be taken into consideration.

In a patient in whom pleural effusion is detected on the chest X-ray, the differentiation between transuda-exuda must primarily be made. The rate of true positivity for exudative fluids is 99.5% and the rate for transudative fluids is 75% in the assessment according to the Light criteria defined by Dr. Richard Light in 1972.[1] Use of the protein gradient along with the Light criteria provides approximately 100% sensitivity and specificity in the differentiation of transuda-exuda.

Many diseases including almost all systems are involved in the differential diagnosis of exudative pleural fluids. However, pneumonia, malignant effusion, tuberculosis and pulmonary thromboembolism are the pathologies comprising the majority of exudative pleural fluids.[2]

Lung cancer, breast cancer and lymphoma are held responsible for development of malignant effusion at a rate of 75%, which is one of the most important causes of exudative pleural effusion. It must be remembered that cancer of any organ can metastasize to the pleura.[2,3]

Although cytological analysis of pleural effusions has a high specificity in the detection of malignant tumors, it has been reported that it has a 30–50% false negativity. It has been suggested that various tumor markers and biochemical parameters can be helpful in the differential diagnosis of difficultly diagnosed malignant effusions. In various studies, carcinoembrionic antigen, beta-2 microglobulin, sialic acid and tissue polypeptide antigen (TPA) have been reported to be high in malignant pleural fluids.[4–6] Reactive oxygen metabolites (ROM) are consistently produced in human. They play a basic role in tissue injury owing to their toxic effects on proteins, lipids, thiols and DNA. Production of ROM increases with the effect of some stimuli such as inflammation, radiation, aging, a higher-than-normal oxygen partial pressure (pO_2_), ozone (O_3_), nitrogen dioxide (NO_2_ ), chemicals and drugs.[7]

There are many studies in which the answer to the following question has been sought: can the increase in ROM pruduction be the cause of any diseases in human? The outcomes of the studies of Petkau[8,9] *et al*. and Breimer[10] suggest that radiation-induced carcinogenesis is initiated with ROM injury. It is suggested that symptoms arising as a consequence of chronic dietary insufficiency of selenium and vitamin E are also exposed by ROM.[11,12] Another condition in which ROM have an important role is aging.[13] Production of ROM increases in the cells of the aging organism resulting in increased lipid peroxidation, lipofuscion production and membrane injury.[14]

In this study, we aimed to investigate whether or not determination of ROM in pleural fluids of patients with malignant and benign pleural effusion can help in the diagnosis, taking into consideration the increasing number of studies on free oxygen radicals in recent years. In this study, ROM levels in serum and pleural fluids of patients with pleural effusion were measured and the levels were compared.

## Methods

In this study, patients with pleural effusion were divided into two subgroups as transudate and exudate according to the Light criteria,[1] and only the cases with exudative pleural effusion were included in the study.

A total of 60 subjects with exudative pleural effusion and 25 healthy subjects as the control group were enrolled in the study. The control group comprised subjects without pleural effusion, pulmonary diseases and/or other systemic diseases assessed by chest X-ray. Of the subjects with pleural effusion, 50% were malignant and 50% were non-malignant [Table 1]. All subjects were informed about the procedure and informed consents were obtained. Ethical approval was given by the hospital commitee.

**Table 1:** Characteristics of the cases

	Cases with malignant effusion	Cases with non-malignant effusion	Control group
Number of cases	30	30	25
Gender			
Male	23 (76.67%)	21(70%)	21(84%)
Female	7 (23.33%)	9 (30%)	4 (16%)
Mean age (years)	51 ± 9	42 ± 14	33 ± 13
Smokers	22 (73.34%)	13 (43.34%)	9 (36%)
Duration of smoking (pack/year)	27.7 ± 13.1	21.0 ± 12.1	12.0 ± 11.1
Mean duration of disease	2 - 7 years	3 - 17 months	
Etiology	Lung cancer [27 (90%)] Lymphoma [2 (6,67%] Ovarian cancer [1 (3.33%]	Pneumonia [21 (90%)] Tuberculosis [7 (23.33%)] Empyema [2 (6,67%)]	

Lung cancer [12 epidermoid carcinomas (44.44%), 4 small cell carcinomas (14.82%), 11 adenocarcinomas (40.74%)] comprised 90% of the malignant cases. Two (6.67%) cases were lymphomas (non-hodgkin) and one (3.33%) case was ovarian tumor (epithelial type). Diagnosis of malignant effusion cases was made by pleural fluid cytology (70%), histopathological analysis of pleural biopsies (23.33%) and pleural biopsy + cytological analysis (6.67%). The amount of pleural effusion was between 500 and 2000 ml in 75% of the patients. While the amount of fluid was below 500 ml in 15% of the cases, there was massive pleural effusion in the remaining 10%. While there was a lymphocyte predominance of 90% in the pleural fluid, eosinophilia (>%10) was detected in 15% of the patients. The mean protein concentration was 4 g/dl, with a range of 1.5 and 8.0 g/dl. The fluids of the patients with malignant effusions were detected as serous in 30% of cases, serousanginous in 40% of cases and hemorrhagic in 30% of cases. The mean erythrocyte count was detected as around 40,000/mm^3^. The erythrocyte count was >100,000 mm^3^ in 15% of cases and pH was below 7.3 in one-third of the cases.

In 12 (57.15%) of the patients in whom pneumonia was detected by chest X-ray and thoracic tomography, the width of fluid was found to be larger than 10 mm, glucose level was >40 mg/dl, pH was >7.2 in pleural fluid analysis and gram staining and culture were negative. In nine (42.85%) cases, the width of the fluid was found to be larger than 10 mm, pH <7.0, glucose < 40 mg/dl, gram staining and culture were positive, and marked pus was not present.

Bacillus tuberculosis was detected in pleural fluids and pleural biopsy samples of cases with tuberculous pleurisy. In the pleural fluid analysis, the pH was between 7.30 and 7.40 and below 7.00 in approximately 20% of the patients. Pleural fluid glucose concentration was above 60 mg/dl in 71.43% of cases and below 30 mg/dl in 28.57% of cases. In the pleural fluid analysis of the patients, there was a slight lymphocyte predominance in more than 50% of the cases.

Although bacteria (*Streptococcus pneumoniae*) were detected on gram staining of pleural fluid in one patient with empyema, bacteria (*Haemophilus influenzae*) were isolated from the pleural fluid culture of the other patient. The same microorganism grew in blood cultures of the patient in whom bacteria were detected in the pleural fluid. The cell number was found to be 42,890 ± 56,816/mm^3^, protein was 4466 ± 1524 g/dl (1.5–8.7 g/dl), glucose was 43.1 ± 39.5 mg/dl (0–96 mg/dl), lactate dehydrogenase was 7851 ± 10503 U/l (378 – 28180 U/l), and the rate for polymorphonuclear leukocytes was found as 90.5 ± 14.5% in the pleural fluid analysis of these patients.

In the study, ROM levels in pleural fluid and sera of the patients with pleural effusion were investigated and only the sera ROM values were investigated in the control group without pleural effusion, as they were healthy individuals.

Simultaneous venous blood and pleural fluid of 10–20 ml were drawn from the cases in the study group and only blood samples were drawn from the control group. Pleural fluid and blood samples were kept at room temperature for 30–60 minutes and centrifuged at 3000–5000 cycles for 10–15 minutes. Thereafter, sera and pleural fluid were separated from the shaped elements using an automatic pipette. The obtained sera and pleural fluid samples were kept in the deep freezer at –35°C in order to study ROM altogether. The ROM kit (Diacron; Grosseto, іtaly) including reactive oxygen radicals (superoxide anion, hydrogen peroxide and hydroxyl radicals) is a commercial kit that provides total measurement of oxidative stress and it was studied with Technicon RA-XT brand autoanalyzer.

### Statistical analysis

Descriptive statistics for the mentioned characteristics were defined as mean, standard deviation, minimum and maximum values. The Student *t*-test and the Mann-Whitney U test were used for determination of the differences between groups in terms of these features. Statistical significance was accepted as 5% in calculations, and the calculations were made using the SPSS (ver. 13) statistical package program.

## Results

**Figure 1:**
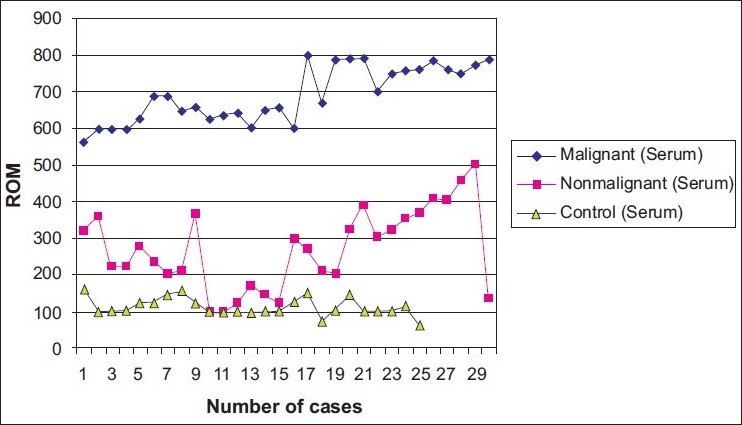
Serum ROM values in malignant and non-malignant pleural effusions and the control group

It was found that ROM values were significantly higher in the malignant group compared to the non-malignant group and the control group, and it was higher in the non-malignant group compared to the control group [Figure 1], and differences between serum ROM values of the three groups were found to be statistically significant (*P* = 0.0001) [Table 2].

**Table 2:** Statistical analysis of serum ROM values in subjects with malignant, non-malignant pleural effusions and the control group

	N	Mean	Standard deviation	Minimum	Maximum	*P*
Malignant group	30	690,0000 a	75,50520	561,00	799,00	0.0001
Non-malignant group	30	269,6667 b	110,03991	98,00	500,00	
Control group	25	110,9200 c	24,76375	60,00	158,00	
Total	85	371,3294	257,80938	60,00	799,00	

**Figure 2:**
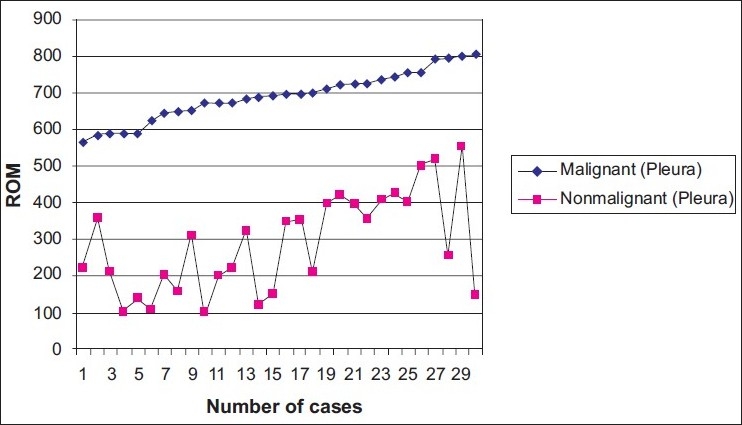
ROM values of pleural fluid in malignant and non-malignant pleural effusions

When ROM values in pleural effusions were compared, it was found that the mean ROM values of malignant effusions were higher than that of the non-malignant group [Figure 2], and the difference was statistically significant (*P* = 0.0001) [Table 3].

**Table 3:** Statistical analysis of pleural ROM values in malignant and non-malignant pleural effusions

	N	Mean	Standard deviation	Standard error	Minimum	Maximum	*P*
Malignant group	30	690,8667	67,81350	12,38100	565,00	810,00	0.0001
Non-malignant group	30	287,4667	134,50439	24,55703	100,00	555,00	
Total	60	489,1667	229,18375	29,58750	100,00	810,00	

In the statistical analysis of pleural and serum ROM values according to 22 (73.34%) smokers and 8 (26.66%) non-smokers in the malignant group, the difference between the two groups was found to be statistically significant (*P* = 0.0001, *P* = 0.004) [Table 4].

**Table 4:** Statistical analysis of pleural and serum ROM values of smokers and non-smokers in the malignant group

	N	Mean	Standard deviation	Minimum	Maximum	*P*
Malignant group (pleural)						0.0001
Smokers	22	698,77	38,462	623	756	
Non-smokers	8	604,00	30,919	565	650	
Total	30	673,50	55,848	565	756	
Malignant group (serum)						0.004
Smokers	22	706,55	69,142	600	799	
Non-smokers	8	624,38	45,907	561	689	
Total	30	684,63	73,051	561	799	

A significant difference was found between the two groups in the statistical analysis of pleural and serum ROM values of 13 smokers (43.34%) compared to 17 non-smokers (56.66%) in the non-malignant group (*P* = 0.0001) [Table 5].

A statistical significance was found between the two groups in the statistical analysis of 9 (36%) smokers compared to 16 (64%) non-smokers in the control group (*P* = 0.0001) [Table 6].

**Table 5:** Statistical analysis of pleural and serum ROM values of smokers and non-smokers in the non-malignant group

	N	Mean	Standard deviation	Minimum	Maximum	*P*
Non-malignant group (pleural)						0.0001
Smokers	13	419,23	66,609	351	555	
Non-smokers	17	184,94	64,846	100	321	
Total	30	286,47	134,535	100	555	
Non-malignant group (serum)						0.0001
Smokers	13	373,92	57,442	300	500	
Non-smokers	17	192,06	60,891	98	298	
Total	30	270,87	108,686	98	500	

**Table 6:** Statistical analysis of pleural and serum ROM values of smokers and non-smokers in the control group

	N	Mean	Standard deviation	Minimum	Maximum	*P*
Control group (serum)						0.0001
Smokers	9	149,44	22,445	123	200	
Non-smokers	16	98,88	13,913	69	123	
Total	25	117,08	30,044	69	200	

## Discussion

Pleural effusion can arise from pulmonary or systemic diseases. Once a pleural fluid sample is obtained, differentiation of transudate-exudate must primarily be made.

Unlike exudative pleural effusions, an increase in reactive oxygen species in the pleural fluid is not anticipated as transudative pleural effusions are not related to local pleural pathologies, and develop related to the association between hydrostatic and oncotic pressures, whereas oxidative stress products increase in diseases causing both malignant and non-malignant exudative effusions as the pleural cavity is involved and mesothelial cells are held responsible for releasing oxidants.[15] ROM in the pleural fluid can have two sources. One of them is passage of increased amounts of plasma and plasma proteins into the pleural space from the pleura via the capillary route; the second can arise from the presence of increased free oxygen radicals in inflammatory cells and malignant cells locally in the pleural space.[16]

Free oxygen radicals are defined as molecular structures including one or more unmapped electrons in their external orbits. Oxidant destruction caused by free oxygen radicals developing as a result of reduction of a single electron plays a role in the pathogenesis of diseases by being present in many events such as ischemia, hyperoxygenation and tissue inflammation.[17,18]

There are many studies investigating the different parameters in the differential diagnosis of malignant and non-malignant pleural effusions. Studies in which tumor markers and tumor growth factors are used are popular. Although the contribution of these parameters to the diagnosis is uncertain, it is reported that combination of cancer antigen (CA 5-9, CA 15-3) and carcinoembryonic antigen (CEA) indicates a probable malignant mesothelioma. CEA is found to be high in malignant pleural effusions related to squamous and adenocarcinoma; CA-3 is found to be high in pleural fluids caused by breast cancer.[19]

In studies conducted with vascular endothelial growth factor (VEGF), it has been reported that this mediator is substantially produced in the diseased pleural tissue and plays a role in pleural fluid development, angiogenesis and vascular permeability, and is found in high amounts in non-small cell lung cancer, mesothelioma and breast cancer.[19]

Insulin-like growth factor values have also been reported to be high in malignant and non-malignant pleural effusions.[20,21]

Recent studies investigating the outcomes of high ROM have established that free radicals are present in the pathophysiology of many diseases. Free radicals are known to be involved in cancer, inflammation injury, aging and chemical toxicity. Along with their effects in carcinogenesis, important roles of oxygen metabolites in inflammation defined as the reaction of live vascularized tissue against local injury have been discussed and depending on this, the increase in their levels is anticipated in diseases resulting in non-malignant effusions.[22]

Our current knowledge is that pleural ROM values increase in non-malignant exudative effusions along with malignant diseases. There are studies in the literature indicating that oxidative stress increases in non-malignant patients. Oxidative stress is reported to increase in active pulmonary tuberculosis, chronic obstructive pulmonary disease, pneumonia, in patients exposed to asbestos and in smokers.[23,24]

In our study, directly ROM in sera and pleural fluid were assessed and ROM values in patients with malignancies, the majority of which comprised lung cancer and patients with non-malignant diseases, were found to be higher than in the control group, consistent with the literature[25–28] (*P* = 0.0001) [Table 3].

Despite the presence of many studies indicating that the serum ROM level increases in patients with malignancies,[25–28] studies indicating that ROM levels increase in malignant effusions are limited.[16,29,30]

We did not encounter a study on the use of pleural fluid ROM values in the differential diagnosis in malignant and non-malignant cases. In our study, we found the pleural fluid ROM values in malignant cases to be significantly higher than that of non-malignant cases (*P* = 0.0001) [Table 3].

There are studies indicating that ROM is affected by smoking, and that ROM levels in smokers are higher compared to non-smokers. In these studies, the increase in ROM in smokers is explained by cigarettes being the source of the oxidants.[31,32] In our cases, both the serum and pleural ROM values of cases with malignant and non-malignant pleural effusion and the serum ROM values of the control group were found to be significantly high in smokers (*P* = 0.0001) [Tables 4‐6].

In conclusion, our study shows that serum ROM values are considerably increased in patients with exudative effusion compared to the control group. This condition suggests that serum ROM values can be instructive in terms of exudative effusion in patients with effusion. Furthermore, detection of significantly high pleural ROM values in malignant pleural effusion compared to non-malignant cases makes ROM appropriate for use as a tumor marker in the differential diagnosis of pleural fluids of unknown origin.
